# An Alternative Data Transformation Approach for ADA Cut Point Determination: Why Not Use a Weibull Transformation?

**DOI:** 10.1208/s12248-021-00625-6

**Published:** 2021-08-13

**Authors:** Gregor Jordan, Roland F. Staack

**Affiliations:** Roche Pharma Research & Early Development (pRED), Pharmaceutical Sciences, Bioanalytical R&D, Roche Innovation Center Munich, Roche Diagnostics GmbH, Nonnenwald 2, 82377 Penzberg, Germany

**Keywords:** ADA, cut point, immunogenicity, transformation, Weibull

## Abstract

The testing of protein drug candidates for inducing the generation of anti-drug antibodies (ADA) plays a fundamental role in drug development. The basis of the testing strategy includes a screening assay followed by a confirmatory test. Screening assay cut points (CP) are calculated mainly based on two approaches, either non-parametric, when the data set does not appear normally distributed, or parametric, in the case of a normal distribution. A normal distribution of data is preferred and may be achieved after outlier exclusion and, if necessary, transformation of the data. The authors present a Weibull transformation and a comparison with a decision tree-based approach that was tested on 10 data sets (healthy human volunteer matrix, different projects). Emphasis is placed on a transformation calculation that can be easily reproduced to make it accessible to non-mathematicians. The cut point value and the effect on the false positive rate as well as the number of excluded samples of both methods are compared.

## INTRODUCTION

Anti-drug antibodies (ADA) measurement is an essential part of the development of protein drugs and is regulated by specific guidelines (1,2). In the scientific community, there has been broad discussion for years about how to calculate a robust cut point (CP) that accounts for biological and technical variability (3–5). The discussion is flanked by the FDA guidelines (2) that require justification for data excluded by outlier testing. Ideally, a data set should retain biological variability (4). Generally, calculation of the CP under the assumption of a normal distribution of the data is a desired goal. However, this is not always possible and using slightly skewed data while assuming a normal distribution is accepted as a “workaround” (3). To actually achieve a normal distribution, data can be transformed (3) (e.g., applying a log transformation) and/or the data set can be tested for outliers (6–9) that are then removed. However, the more data that is excluded (assuming these were not caused by pre-existing ADA (pADA)), the greater the risk of observing an artificially high false positive rate (6) in the study samples because the recommended 5% false positive rate (2) is calculated based on the remaining data points. This could make an in-study CP necessary (3,10, 11) which is not considered optimal, as it could slow down data reporting although it is however scientifically sound. The impact of the in-study cut point has to be fully assessed before implementation (4) taking challenges into account that in some rare disease clinical trials, only a limited number of patient are enrolled.

One possible way to address a non-normal distribution is to perform a suitable transformation of the data to fit the skewed distribution. Skewed distributions are often found in survival analysis, extreme value theory, weather forecasting, reliability engineering/failure analysis (12), and oncology data (13). In the case of ADA assays, a skewed distribution may result for technical reasons if an assumed normal distribution of the samples is obscured by assay conditions that compress sample signals at the lower end of the distribution. A Box-Cox transformation can bring skewed data sets into a normal distribution and the commonly used log transformation is a special case of the Box-Cox transformation (14). The use of a Weibull transformation offers a promising alternative (12,15,16). Estimation of the unknown parameters could be done according to the maximum likelihood method. As this approach is not easy to perform for inexperienced mathematicians, we present a pragmatic approach. An Excel®-based method was chosen deliberately because this software is widely used and the Excel add-in Solver can be used to determine the value of unknown variables in the Weibull equation. We lay out this simplified approach to start a discussion within the bioanalytical community based on the proposed transformation procedure.

## METHODS

### Weibull Transformation

Outliers were removed from the normalized screening assay data set using the 3IQR rule (7) with the ulterior motive of minimal data manipulation according to the guideline (2). The remaining data were transformed using Equation 1 (12,15). Normalization was performed by dividing each individual signal by the signal of a pool matrix on each plate.

1 $$ f(x)=\left(a/b\right)\ \left(x/b\right)\hat{\mkern6mu} \left(a-1\right)\ \exp \left(-\left(x/b\right)\hat{\mkern6mu} a\right) $$

In Equation 1, *a* is the shape parameter, *b* is the scale parameter, and both are unknowns and must be determined to transform the data set. Our method leverages the Excel SKEW function and the Excel (Microsoft) add-in “solver” to do so. The skewness value of the data set is determined using the Excel SKEW function and then driven as close to zero as possible by adjusting the *a* and *b* parameters of the transformation equation using the Solver add-in. The Solver settings were defined such that the unknown parameters *a* and *b* remained in the range 0.01–100. The Solving Method in Solver was set to “GRG Nonlinear.” All transformed data were plotted against the untransformed data to confirm that the ranking did not change randomly.

All data sets (non-transformed and transformed) were subjected to a Shapiro–Wilk test in R (shapiro.test, R 3.5.1, www.https://www.r-project.org/) to confirm normal distribution.

### Decision Tree-Based Calculation

This Weibull transformed data were compared with results obtained with an approach based on the following decision tree: The original data were checked for normal distribution. In the case of a non-normal distribution, a log-transformation was performed and tested for normal distribution. The normal distribution was verified using the shapiro.test, or if the *p*-value was <0.05, normality was assumed if the skew factor (SKEW, Excel) was <ABS (1)(3). In the case that the distribution was still estimated to be non-normal, the data set was subjected to a 3IQR outlier test and then analyzed for normal distribution (as described above, including the log transformation). In case of a still non-normal distribution (via shapiro.test or skewness), the data set was subjected to a 1.5 IQR outlier test and the normal distribution was tested again. The CP was calculated with a parametric approach based on the data set that first achieved normal distribution. If no normal distribution was achieved, the CP was calculated based on the 95th percentile of the 1.5 IQR data.

To enable a direct comparison of the CP factor of all data sets, regardless of whether they were determined from transformed or untransformed data sets, the CP factor of log and Weibull transformed data sets were re-transformed. For Weibull transformed data, the back calculation was performed by using the Excel add-in “solver” tool.

The data sets from validation or pre-validation runs (blank healthy volunteer matrix) of 10 different projects (monoclonal antibodies and multidomain therapeutics) using ADA one-step bridging assays (with drug as capture and detection reagent) were transformed by both methods and then compared. The number of individual matrix samples used for the evaluation is shown in Table I and covers a sample size from 40 to 128 individuals. Variability of the assay (replicate assay runs) was not in the scope of the assessment. In order to assess the applicability of the introduced Weibull-based approach to data sets with replicate variability, the transformation was assessed for two data sets were three replicates of 100 individual matrix samples were analyzed on different days by different operators.

**Table I:**
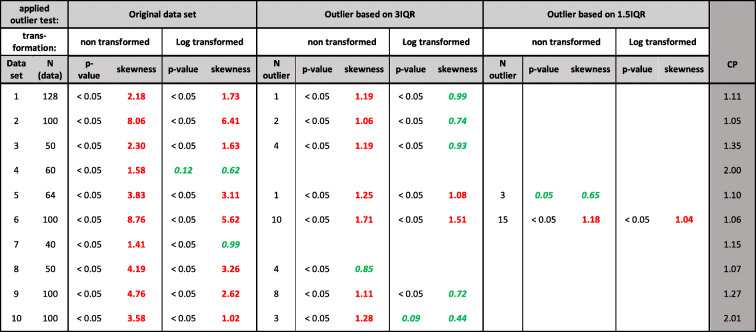
CP Calculation for 10 Data Sets by Applying Decision Tree of Fig. 1. Red Bold Value: Indicating Non-normal Distribution. Green Italic Value: It Is Assumed that a Normal Distribution Exists and Data Set Was Selected for CP Calculation. *p*-value Based on Shapiro–Wilk Test in R. Number of Detected Outliers for Each Applied Test Is Shown in “N Outlier.” In Case No Normal Distribution Can Be Assumed, the CP Is Calculated Based on 95 Percentile; Otherwise, a Parametric Approach Was Selected

## RESULTS

**Fig. 1:**
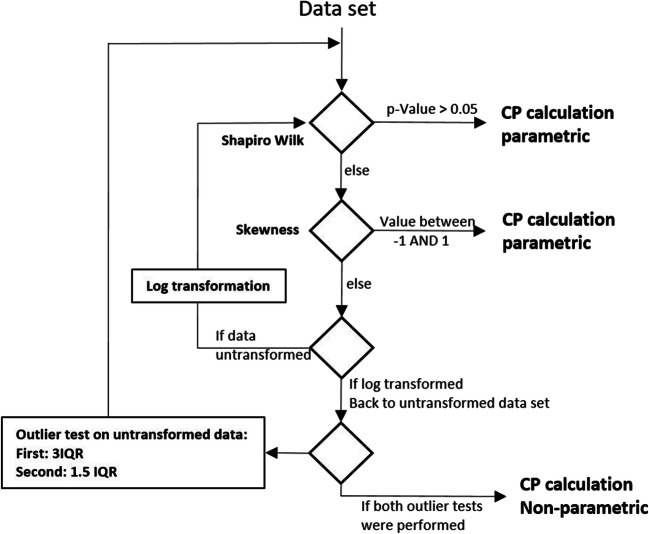
Cut point calculation procedure for decision tree CP calculation

Table I summarizes the CP calculation based on the decision tree, non-Weibull transformation approach. Two data sets were normally distributed after log transformation, 6 data sets had to undergo 3IQR outlier tests, and two data sets underwent 1.5 IQR. The CP was calculated based on the criteria described in Fig. 1.

**Fig. 2:**
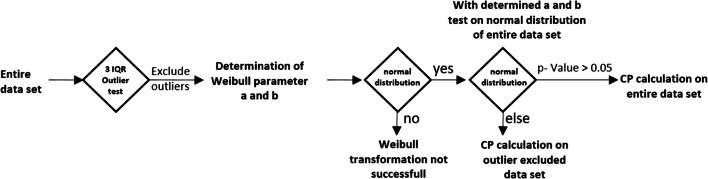
Flow chart for the Weibull normalization approach

Table II summarizes the Weibull-transformed data. All data sets could be transformed into a normal distribution with *p*-values in the range of 0.05 to 0.95. For further evaluation, the calculated values *a* and *b* were retained for each data set, a new transformation based on the complete data (without outlier exclusion), and the *p*-value was calculated (Fig. 2).

**Table II:** CP Calculation by Applying the Weibull Transformation. Parameters *a* and *b* Were Determined Based on the Data Set Which Were Undergoing the 3IQR Outlier Test. Keeping a and b, the Test on Normal Distribution Is Repeated Based “on All” Data, and in Case of a Normal Distribution, the CP Was Re-calculated. Presence of Potential pADA Based on Quenching Signal in Confirmatory Assay

	Outlier based on 3IQR				CP (based on 3IQR)	All data	CP (on al)
Data set	*N* outlier	a (shape)	b (scale)	*p*-value		*p*-value	
1	1	0.80	0.09	**0.80**	1.12	0.61	1.13
2	2	0.68	0.09	**0.54**	1.06	< 0.05	
3	4	0.71	0.10	**0.19**	1.45	0.09	1.94
4	1	0.43	15.0	**0.83**	1.99	0.77	2.07
5	1	1.03	0.10	**0.27**	1.16	0.41	1.16
6	10	2.49	0.50	**0.05**	1.11	< 0.05	
7	0	0.66	0.10	**0.95**	1.17		
8	4	0.69	0.10	**0.07**	1.08	< 0.05	
9	8	1.20	0.36	**0.17**	1.36	0.05	1.81
10	3	0.92	0.74	**0.23**	2.17	0.27	2.44

The aim was to evaluate the robustness of the Weibull transformation and to evaluate whether the extreme outliers were part of the distribution. In principle, the data set should only be subjected to minimal manipulation and outlier removal should be justified (2), for example, by the presence of pADA or bivalent soluble target (17). By applying the transformation on the complete data sets, the *p*-values of the data sets 2, 6, and 8 fell below 0.05, whereas all others remained above 0.05. Data sets 2, 3, 6, 8, 9, and 10 had more than 2 outliers based on the applied 3IQR test. The data sets 2, 6, and 8 required outlier removal for applying the Weibull transformation (Table II). In these data sets, the outliers showed a significantly higher quenching signal (data not shown) compared to the mean. This supported the legitimacy of their removal as they appeared to be more reactive (e.g., due to pADA or drug target) than most other samples to the drug. In contrast, for the remaining data sets (3, 9 and 10), the *p*-value fell not below 0.05 (Table II) when Weibull parameters were applied to the entire data sets, enabling the entire dataset to be used for cut point determination. As long as the *p*-value of the entire data set remains above 0.05, a CP can be calculated based on the entire data set assuming the 3IQR test eliminated erroneous values. A comparison of the CP is shown in Table II where, when applicable, the CP is based on the entire data set.

Table III summarizes a comparison of the two approaches in terms of CP and false positive rate. Additionally, the false positive rate is calculated using the Weibull-derived CP for the entire data set when applicable. In theory, the false positive rate should be at 5%, thus, a robust CP determination method should deliver a value as close to 5% as possible.

**Table III:**
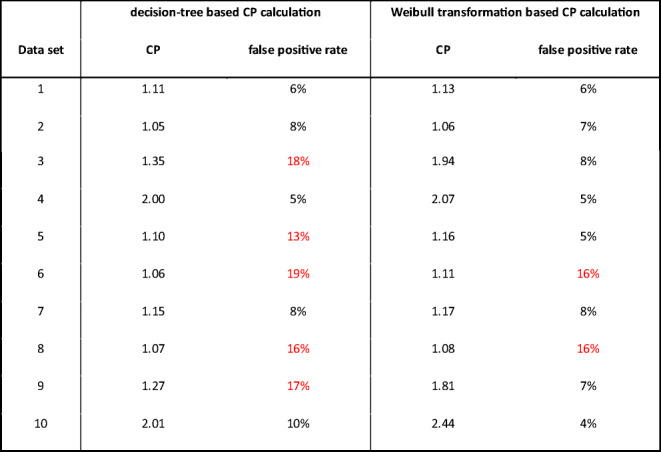
Comparison of CP Calculation Based on Assumption of a Weibull Distribution and Decision Tree (Fig. 1). False Positive Rate Is Based on Applying Each CP on the Complete Data Set. Red Values Were Outside the Range of 2 to 11% False Positive Rate

The robustness of the Weibull transformation was additionally evaluated on data sets 2 and 9 for which 3 replicates of the 100 individual samples were performed on different days by different analyst, resulting in *N*=300 data sets. After outlier removal (3 IQR), the data sets could be transformed to normal distribution by applying the Weibull approach with *p*-values of 0.40 and 0.24. For data set 2, the initial (based on *N*=100) determined values *a* and *b* could be kept and the *p* value dropped from 0.54 (*N*=100) to 0.40 (*N*=300).

## DISCUSSION AND CONCLUSION

We have introduced a Weibull-based transformation for screening assay data based on a procedure that can be easily repeated and reproduced by bioanalytical experts. The applicability of the transformation was demonstrated for 10 data sets, and a comparison with a decision tree-based CP calculation method highlighted the advantages of the introduced Weibull transformation in terms of (a) transformation of data to normal distribution and (b) a more robust CP calculation that reduces the need to exclude outliers, additionally its potential sensitivity in detecting outliers as potential reactive samples (e.g., pADA, soluble target). The reduced number of excluded data points for which there would be no justification such as pADA shows a major advantage and meets regulatory expectations (2). Ideally, the assessment of screening reactive samples for the presence of pADA should be based on a dedicated assay (e.g., IgG depletion) and not only on the confirmatory assay results (17). In a comparison of the false-positive rate, the Weibull transformed data were superior to results from the decision tree-based CP approach. The overall values were close to the theoretical 5% level, which suggests that an in-study CP might not be necessary (in case a nearly representative population was chosen for CP calculation). The CP of 8 out of 10 data sets were within 2–11% (3,10) with the Weibull transformation, whereas only 5 were in that range with the decision tree-based CP approach. A recent publication highlighted that the false positive rate is related to the number of samples included in the CP calculation (11). Therefore the expected false positive rate for 100 data points for data set 10 should rather be between 2 and 9%. Data set 2 suggested a more robust CP with the Weibull transformation compared to the decision tree-based approach given that low CP values might result in a high false positive rate during routine sample analysis. However, the difference is low or even negligible. For data sets 2 and 9 for which 3 replicates resulting in N=300 data sets were available, the Weibull-based transformation could be applied as well, showing its applicability also for pooled data sets coming from replicate assay runs.

Taken together, the Weibull transformation of a screening assay results and process described in Fig. 2 provides an alternative way to incorporate more data into a normally distributed transformed data set, is sensitive enough to enable the detection and removal of pre-existing drug reactive samples, and can achieve the goal of setting a cut point that results in a 2–11% false positive rate.

It is clear to the authors that the screening results should be evaluated in light of the confirmatory assay (robust elimination of pADA) but it may be difficult to define a criterion in the confirmatory assay that detects potentially pADA-containing samples without an additional assay like IgG/IgM depletion. A robust screening CP calculation, as presented, characterized by the need to exclude a smaller number of data points could also be beneficial for confirmatory assay assessment, as the focus would only be on a smaller number of samples and a significant quenching might be clearer. Nevertheless, the approach presented was chosen to focus on the robustness of the transformation procedure and on the transformation itself, based on screening assays, with the aim to cope with regulatory expectations in terms of minimal data manipulation. Other tools (e.g. R) could be used to generate more statistically sound calculations of the scale and shape parameters, but were beyond the scope of this analysis.

Finally, a re-analysis of complete assay validation data sets, e.g, recommended by the FDA (2) and clinical data where an in study cut-point was necessary using the Weibull approach could demonstrate its applicability. The presented approach could open new possibilities for data transformation for ADA immunoassays and re-evaluation.
